# Public Adverse Event Data Insights into the Safety of Pembrolizumab in Melanoma Patients

**DOI:** 10.3390/cancers12041008

**Published:** 2020-04-19

**Authors:** Anne Schaefer, Christos Sachpekidis, Francesca Diella, Anja Doerks, Anne-Sophie Kratz, Christian Meisel, David B. Jackson, Theodoros G. Soldatos

**Affiliations:** 1 Molecular Health GmbH, 69115 Heidelberg, Germany; 2 Clinical Cooperation Unit Nuclear Medicine, German Cancer Research Center (DKFZ), 69120 Heidelberg, Germany; 3 Department of Nuclear Medicine, Inselspital, Bern University Hospital, University of Bern, 3010 Bern, Switzerland

**Keywords:** Pembrolizumab, melanoma, immune checkpoint inhibitors, adverse events, FDA’s Adverse Event Reporting System (FAERS), VigiBase, pharmacoepidemiology, data mining

## Abstract

Immune checkpoint inhibition represents an important therapeutic option for advanced melanoma patients. Results from clinical studies have shown that treatment with the *PD-1* inhibitors Pembrolizumab and Nivolumab provides improved response and survival rates. Moreover, combining Nivolumab with the *CTLA-4* inhibitor Ipilimumab is superior to the respective monotherapies. However, use of these immunotherapies is frequently associated with, sometimes life-threatening, immune-related adverse events. Thus, more evidence-based studies are required to characterize the underlying mechanisms, towards more effective clinical management and treatment monitoring. Our study examines two sets of public adverse event data coming from FAERS and VigiBase, each with more than two thousand melanoma patients treated with Pembrolizumab. Standard disproportionality metrics are utilized to characterize the safety of Pembrolizumab and its reaction profile is compared to those of the widely used Ipilimumab and Nivolumab based on melanoma cases that report only one of them. Our results confirm known toxicological considerations for their related and distinct side-effect profiles and highlight specific immune-related adverse reactions. Our retrospective computational analysis includes more patients than examined in other studies and relies on evidence coming from public pharmacovigilance data that contain safety reports from clinical and controlled studies as well as reports of suspected adverse events coming from real-world post-marketing setting. Despite these informative insights, more prospective studies are necessary to fully characterize the efficacy of these agents.

## 1. Introduction

The discovery of immune checkpoints and their role in anti-tumor immunity has revolutionized treatment of metastatic melanoma. The key targets of immune checkpoint inhibition include the cytotoxic T-lymphocyte-associated protein 4 (*CTLA-4*) and the programmed cell death protein 1 (*PD-1*), both of which are expressed by T cells. Binding of *CTLA-4* to *CD80*, which is expressed by antigen presenting cells, impairs T cell activation, whereas binding of *PD-1* to *PD-L1*, which is expressed by epithelial cells, immune cells, and tumor cells, induces anergy and exhaustion of the activated T cell [1,2,3]. Thus, the immune checkpoint blockade enhances anti-tumor immunity by increasing T cell activation and preventing their inhibition [4].

Ipilimumab, a monoclonal antibody targeting *CTLA-4*, was the first checkpoint inhibitor that in patients with advanced melanoma demonstrated an improvement in overall survival (OS). In 2011, it was approved by the United States (US) Food and Drug Administration (FDA) for the treatment of unresectable or metastatic melanoma [5]. A few years later, trials with antibodies inhibiting the *PD-1* pathway demonstrated superiority to chemotherapy in Ipilimumab-refractory melanomas, resulting in the approval of Pembrolizumab and Nivolumab for the treatment of refractory unresectable or metastatic melanoma in 2014 [6,7,8,9,10]. Additionally, Pembrolizumab was shown to improve OS, response rate and progression-free survival (PFS) compared to Ipilimumab in patients with previously untreated advanced melanoma in the randomized, controlled, phase 3 KEYNOTE-006 study [11,12]. Moreover, superiority of Pembrolizumab was confirmed by the results of the recent long-term five-year follow up analysis of patients within this study, revealing median OS of 32.7 months for patients that received Pembrolizumab compared to 15.9 months for patients treated with Ipilimumab [13]. Furthermore, given the non-redundant functions of *CTLA-4* and *PD-1*, the rationale to combine anti-*CTLA-4* and anti-*PD-1* antibodies was tested in the CheckMate 067 trial. Indeed, the combination of Nivolumab with Ipilimumab was superior to Ipilimumab and to Nivolumab monotherapy, with PFS of 11.5, 2.9, and 6.5 months, respectively [14].

According to the positive results from these studies, treatment with anti-*PD-1* antibodies—as mono- or combination-therapy with Ipilimumab—is nowadays a major therapeutic option for advanced melanoma patients with good performance status [15]. However, despite the success of immune checkpoint inhibitors (ICI) in melanoma treatment, approximately 40% to 45% of patients experience no response to therapy. Moreover, administration of these agents is associated with the emergence of a ‘new class’ of side effects, collectively referred to as immune-related adverse events (irAEs). These cumulative, dose-dependent, and sometimes life-threatening immune-mediated toxicities can theoretically affect any organ and are of inflammatory character [16], thereby reflecting the immune checkpoints’ role in regulating adaptive immunity. In patients treated with ICI monotherapies, reported incidences of any grade irAEs range from 15% to 90% [17] and mainly affect the gastrointestinal tract, the liver, the endocrine glands, and the skin [16]. Nonetheless, depending on the type of inhibitor, some irAEs are more frequently observed than others [18], with lower rates of high-grade irAEs among anti-*PD-1* antibodies compared to anti-*CTLA-4* antibodies. Importantly, as the combination of anti-*PD-1* and anti-*CTLA-4* leads to a more enhanced immune activation, the frequency of high-grade irAEs for the combination therapy is higher than for either monotherapy [14,19].

Early recognition of these irAEs is very important and the discontinuation of immunotherapy and administration of corticosteroids is recommended for successful management [20]. However, the biomolecular landscape underlying ICI therapy is not yet fully characterized and biomarkers are necessary for predicting response, resistance and/or toxicity in a systematic way. Since the effective management of irAEs relies mainly on their early recognition [21,22], several recent efforts emphasize the need for more evidence-based data and for the identification and characterization of molecular biomarkers and the genomic correlates of ICI response and irAE toxicities [23,24,25,26,27,28].

The aim of our study is to utilize real world evidence to provide additional insights to these profiling efforts of ICIs, and of Pembrolizumab in particular, in the context of melanoma treatment. We, therefore, searched for sources of accessible clinical phenotype data, such as adverse events (AEs), and summarized a body of data encompassing more patients than examined in other clinical trials or translational studies reported thus far. For our analyses, we relied on data coming from two different AE reporting systems (AERS).

Specifically, we processed AE data from the US FDA AERS (FAERS)—including the legacy AERS (LAERS) data—and the VigiBase repositories. FAERS contains AE reports, medication error reports and product quality complaints resulting in AEs that were submitted to the FDA. This computerized information system is designed to support the US FDA’s post-marketing safety surveillance program. VigiBase is maintained by the World Health Organization (WHO) and contains global individual case safety reports submitted by the participating member states enrolled under the WHO’s international drug monitoring program. FAERS and EudraVigilance from the European Medicine’s Agency constitute the main AE reporting management and evaluation systems in the US and Europe, respectively, while VigiBase is the single largest drug safety data repository in the world. Our findings are intended to contribute to the emergent knowledge about irAE’s and ultimately facilitate more informed ICI therapy monitoring.

## 2. Materials and Methods 

**Figure 1:**
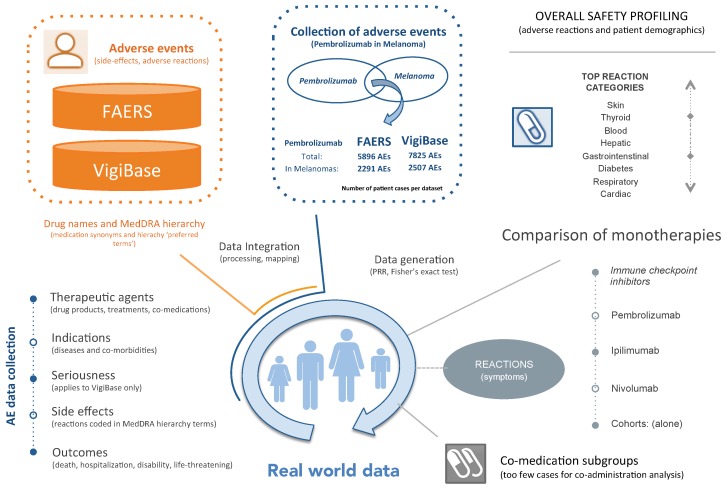
Synopsis of our study: To review adverse events (AEs) related to the therapy of melanoma patients with Pembrolizumab, we integrated and standardized AE data from two public repositories (FAERS and VigiBase). From each repository, we extracted a separate cohort of AEs that contained melanoma patient cases treated with Pembrolizumab. Using disproportionality metrics, these cohorts were retrospectively characterized with respect to demographics, outcomes, side effects and adverse reactions reported for those patients. In addition, we report on reaction classes listed in FAERS AEs that mention only key ICIs (namely, Ipilimumab, Nivolumab, and Pembrolizumab), without any other co-medications. Last, we expect that our findings as observed in these datasets add to the combined efforts to more effectively monitor and react on irAEs.

To examine the safety of Pembrolizumab in the treatment of melanoma, we reviewed public AE patient data extracted from the FAERS and VigiBase repositories. The datasets included AE data released for 2017Q2 by FAERS and for 2018Q2 by VigiBase. Finally, we characterized the safety of Pembrolizumab by examining AE information as observed in those datasets for melanoma patients. Figure 1 summarizes our approach.

### 2.1. Adverse Event Data Integration

We processed 7.9 million cases (9.5 million reports) from the publicly available FAERS data set and 17 million case reports from VigiBase. Both datasets contain de-identified AE patient data and are similar in that they hold records with information about the patients’ drug therapies, the indications for those drugs (disease or condition), and the observed reactions and outcomes (e.g., “death” or “hospitalization”) reported in these AEs.

One challenge in the computational processing of data from FAERS is the unambiguous identification of the therapeutic agents relevant to an AE that may be submitted by reporters in free text descriptions (e.g., “PEMBROLIZUMAB, 25 MG/ML”). While such names may be easily understandable by humans, they may also contain spelling and typographical errors, language variations, multiple medications in one phrase, as well as irrelevant terms. To resolve such ambiguities introduced by the non-standardized use of drug names [29], FAERS free-text medication descriptions were consolidated and matched to standardized drug synonyms compiled from DrugBank and PubChem records [30]. In VigiBase, the medications reported for each AE are structured in a different way, organized by drug product name and ingredient substances.

In comparison, reactions are coded by both datasets in terms from the Medical Dictionary for Regulatory Activities (MedDRA) and they were analyzed at the Preferred Term (PT) level of the hierarchy (i.e., MedDRA Level 4 descriptions).

### 2.2. Definition of Cohorts

We identified 5896 and 7825 AE patient cases from FAERS and VigiBase, respectively, that referred to treatment with Pembrolizumab in any disease. These AEs were defined as FAERS cases that were annotated with the ‘Pembrolizumab’ drug record, or as VigiBase cases with product (drug) names mentioning ‘Pembrolizumab’, ‘Keytruda’, or ‘Lambrolizumab’. Using these AEs we searched for melanoma-specific subsets, and defined the two main cohorts that were analyzed:

- FAERS PembroM: 2291 AE cases from FAERS of melanoma patients treated with Pembrolizumab.
- VigiBase PembroM: 2507 AE cases from VigiBase of melanoma patients treated with Pembrolizumab.

The two sets (FAERS PembroM and VigiBase PembroM) were processed separately, as VigiBase contains AEs that are also reported in FAERS. Each cohort was examined with respect to reported demographics, drugs, indications, reactions, and outcomes. Melanoma cases were defined to be those for which reported indications linked to the MedDRA hierarchy term ‘Skin melanomas (excluding ocular)’, such as ‘MALIGNANT MELANOMA’, ‘METASTATIC MALIGNANT MELANOMA’, or ‘MALIGNANT MELANOMA STAGE IV’. 

Both cohorts were also examined for reactions mentioned in AEs of melanoma patient cases reporting Pembrolizumab as single treatment. These AE subsets were named ‘Pembrolizumab (alone)’.

### 2.3. Statistical Characterization

For the statistical characterization of the cohorts, we utilized the proportional reporting ratio (PRR) score, an established measure of disproportionality in pharmacovigilance [31,32] In our analysis, PRR was defined as the value of *a*(*c* + *d*)/*c*(*a* + *b*) from the following contingency matrix (1).


**AE Cases**

**Event (E)**

**Not E**

**Totals**

**Cohort**

*a*

*b*
*a* + *b*
**Not Cohort**

*c*

*d*
*c* + *d*Totals*a* + *c**b* + *d**N* = *a* + *b* + *c* + *d*

An event E may represent the occurrence of reaction(s), drug(s), indication(s), or of patient outcome(s) in the AEs of each inspected Cohort, e.g., FAERS PembroM or VigiBase PembroM. To systemize the calculations, each cohort’s AEs were compared against the whole set of remaining cases from the respective dataset, FAERS or VigiBase. This means that the comparative ‘Not Cohort’ background contained all other AEs from each repository. For instance, in the case of the PembroM cohorts, the background may include AEs that contain other melanoma cases or cases that may have reported Pembrolizumab but not both melanoma and Pembrolizumab, because these were included in the PembroM cohort by definition. In turn, this may have resulted in less strong signals per event E in each cohort but more specific. Last, we also considered statistical significance be indicated by a Fisher’s exact test *p*-value lower than 5% (i.e., when *p*-value < 0.05).

### 2.4. Data Availability

The Supplementary file lists results for the FAERS PembroM and the VigiBase PembroM cohorts. The file summarizes also demographic information, as well as gender distribution for each reaction, as observed in each of these two cohorts. Last, the supplementary file includes observed data for each reaction reported in the examined ‘Pembrolizumab (alone)’ cohorts, too.

## 3. Results

**Figure 2:**
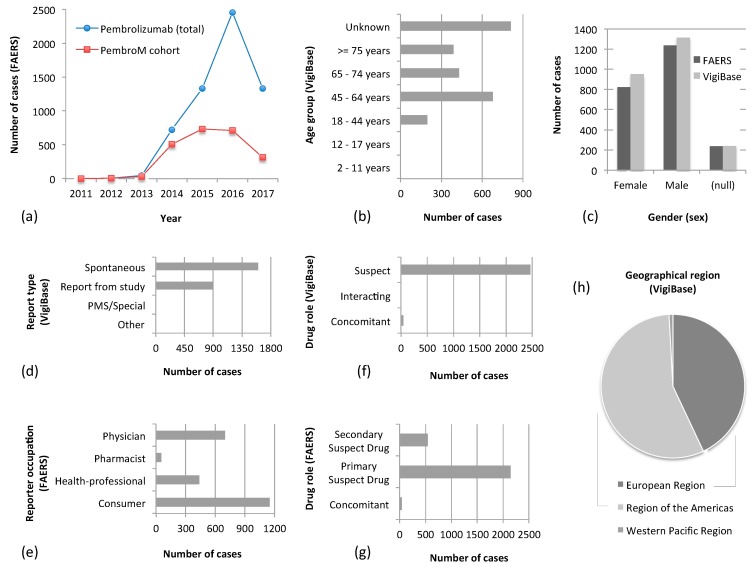
Demographics of examined adverse event (AE) cohorts. The table/figure summarizes (**a**) distribution of all Pembrolizumab AEs in FAERS and of FAERS PembroM AEs, over time; the distribution over time does not include incomplete AEs for which no date was specifically registered; AEs dated prior to Pembrolizumab’s approval in 2014 may reflect reports from preapproval studies and clinical trials. The reduction in AE numbers for 2017 is explained by the fact that the full dataset for that year was not yet released by FAERS at the time that data were gathered; (**b**) Distribution of VigiBase’s PembroM melanoma patient ages—most cases were reported for the age group between 45 to 64 years; (**c**) Sex distribution in PembroM FAERS and VigiBase cohorts—both cohorts contain more patients of male gender; (**d**) Distribution of report types submitted to VigiBase—most AE reports are spontaneous; (**e**) Distribution of reporters’ occupations in FAERS—submitted information about patient cases came both from expert health professionals (physicians, pharmacists, other) but also directly from consumers in FAERS; (**f**) Distribution of Pembrolizumab’s ‘role’ in VigiBase’s PembroM cases—in most cases, Pembrolizumab was considered as the ‘suspect drug’ causing the observed AE and only twice was it reported as ‘interacting’; (**g**) Distribution of Pembrolizumab’s ‘role’ in FAERS’s PembroM cases—as in the case of VigiBase, Pembrolizumab was considered as either the primary or the secondary ‘suspect drug’ responsible for observed AE reactions; (**h**) VigiBase PembroM AE reports come mainly from the regions of Europe and the Americas.

The data extracted from FAERS and VigiBase are complementary although there is some degree of overlap. Figure 2 highlights some such examples.

One main difficulty that regulatory authorities face when gathering AE safety data is the systematic and consistent representation of suspected AE case reports that may come with a variety of detail, forms and quality. This may change over time and may be due to a multitude of different circumstances. In turn, features such as age or gender may be missing or highlighted as ‘unknown’ (Figure 2b,c) as well as other important clinical information (e.g., dosage, administration route, or duration of symptoms) may be sparse or even unavailable. These collection and reporter/submitter implications are aspects that each spontaneous repository cannot effectively control and handles them similarly in scope, but also differently in terms of chosen data representations or levels of abstraction. For example, VigiBase provides annotations on the ‘report type’ (Figure 2d) whereas FAERS on the ‘reporter’s occupation’ (Figure 2e). The suspected drug’s ‘role’ is handled somewhat differently (Figure 2f,g) in that FAERS specifies whether a reported drug was ‘primary’ or ‘secondary’ suspect, whenever such information is available.

This is important information to consider as in our study several cases were dated prior to Pembrolizumab’s approval and approximately up to 36% of VigiBase PembroM cases were based on reports annotated as ‘coming from studies’. It is reasonable to assume that patients evaluated in some of these cases were enrolled in clinical trials, closely controlled, and followed using determined protocols and intensive follow-up regimens, but this (or which ones exactly) cannot be confirmed from these data alone. Therefore, we did not exclude them from our analysis and handled them uniformly with other real-world post-marketing data, occurring outside controlled and/or clinical trial environments.

Further complications may rise when dealing with AE data considering that for some cases multiple reports may be available over time, sometimes duplicates, and sometimes with conflicting information. Such aspects are tackled by each AERS internally, and are very difficult to handle by examining only the publicly available, de-identified data. However, some such ‘discrepancies’ may be explicable, for example due to the fact that one drug may be assigned different ‘roles’ (e.g., suspect or concomitant) for the same AE (depending on which observed reaction is referred to each time), or because patient-case information may be updated (or corrected) with follow-up reports. In that respect, FAERS and VigiBase handle their data somewhat differently, partially attributed to FAERS receiving sometimes multiple reports for the same case from different submitters whereas VigiBase gathers individual safety records directly from national authorities, including content coming from FAERS. 

To avoid processing the same cases multiple times, in our study we analyze and present results for each PembroM cohort separately (once for FAERS and once for VigiBase). Each shared event is therefore counted only once in each cohort, and individual data set characteristics and intricacies (complications and advantages) were neither combined nor compromised. 

### 3.1. Overview of the PemboM Cohorts

In total, our AE dataset held 2291 FAERS cases (FAERS PembroM cohort) and 2507 VigiBase cases (VigiBase PembroM cohort) of melanoma patients treated with Pembrolizumab. Figure 2 summarizes key demographic characteristics of these cohorts. Among these, 61.2% of the FAERS cohort (1402 AEs) and 66.2% of the VigiBase cohort (1660 AEs) reported only Pembrolizumab and no other drugs. We named these (sub-) cohorts ‘Pembrolizumab (alone)’.

Furthermore, representation of other ICIs in the PembroM cohorts was sparse—Ipilimumab was mentioned in 114 FAERS and in 91 VigiBase AEs (in 59 AEs with product name ‘Ipilimumab’ and in 32 as ‘Yervoy’), Atezolizumab was mentioned in three FAERS and in three VigiBase AEs (in two cases with product name ‘Atezolizumab’ and in one as ‘Tecentriq’), whereas Nivolumab was reported in nineteen FAERS and in 23 VigiBase (sixteen times as ‘Nivolumab’ and seven as ‘Opdivo’) cases.

Among the most frequent non-melanoma related co-morbidities reported in the two PembroM cohorts, there were several terms that referred to the general physical condition of the patients in addition to very specific and irAE-related symptoms. Characteristically, the twenty most frequent non-melanoma-related indications mentioned in either of the two cohorts included the following MedDRA PTs: ‘hypertension’, ‘pain’, ‘hypothyroidism’, ‘prophylaxis’, ‘constipation’, ‘product used for unknown indication’, ‘routine health maintenance’, ‘nausea’, ‘gastroesophageal reflux disease’, ‘anxiety’, ‘diabetes mellitus’, ‘hypercholesterolaemia’, ‘pruritus’, ‘insomnia’, ‘depression’, ‘rash’, ‘hyperlipidaemia’, ‘diarrhoea’, ‘arthralgia’, ‘cough’, ‘hypertension arterial’, ‘atrial fibrillation’.

Melanoma-related indications included a variety of terms, the most frequent ones being ‘melanoma’, ‘metastatic melanoma’, ‘metastatic malignant melanoma’, ‘malignant melanoma stage iv’, ‘malignant melanoma stage iii’, and ‘melanoma recurrent’. While this suggests that many patients suffered severe melanoma conditions, in the absence of additional (clinical or historical) data it could not be clarified whether (or which) PembroM cohort cases possibly came from the treatment of non-resectable advanced melanoma or from resected melanoma cases that were adjuvantly treated. The term ‘adjuvant therapy’ was reported only once in each cohort, possibly reflecting the same case.

Overall, the FAERS PembroM cohort captured more cases with severe patient outcomes (death, hospitalization, life-threatening, disability/incapacitating) than the VigiBase PembroM cohort, and 70.2% of the latter cohort’s cases were explicitly annotated as ‘serious’ (1760 AEs).

### 3.2. Adverse Event Profiling

We extracted side effect profiles for the two PembroM cohorts by summarizing adverse reactions described in MedDRA PTs (i.e., level 4 terms) that were reported in FAERS and VigiBase. The following PTs were not considered, whenever mentioned: ’Adverse event’, ‘Death’, ‘Disease progression’, ‘Inappropriate schedule of drug administration’, ‘Malignant neoplasm progression’, ‘Metastatic malignant melanoma’, ‘Metastases to central nervous system’, ‘Malignant melanoma’, ‘Toxicity to various agents’, ‘Therapy non-responder’, ‘Therapy partial responder’.

The two datasets were consistent with respect to the types of adverse reactions they contained. Table 1 juxtaposes occurrence of reactions that were mentioned in both PembroM cohorts with the same MedDRA PTs, while Table 2 summarizes our findings altogether in groups of key organ/system categories and denotes which PembroM cohort they were derived from.

Overall, our analysis could re-capitulate several MedDRA PT reactions that likely reflected irAEs, among which some with high PRR signals (>5), such as:

- ‘Hypophysitis’ (PRRs: FAERS = 267.82; VigiBase = 308.83) and ‘Adrenal insufficiency’ (PRR: FAERS = 28.10; VigiBase = 47.47)
- ‘Vitiligo’ (PRRs: FAERS = 166.64; VigiBase = 373.83), ‘Pemphigoid’ (PRRs: FAERS = 24.48; VigiBase = 53.54), and ‘Rash maculo-papular’ (PRR: FAERS = 9.53)
- ‘Autoimmune colitis’ (PRR: VigiBase = 565.03) and ‘Colitis’ (PRRs: FAERS = 35.8; VigiBase = 22.1)
- ‘Thyroiditis’ (PRRs: FAERS = 42.42; VigiBase = 81.45), ‘Hypothyroidism’ (PRRs: FAERS = 11.22; VigiBase = 30.35), ‘Hyperthyroidism’ (PRRs: FAERS = 7.16; VigiBase = 23.94), and ‘Thyroid disorder’ (PRRs: FAERS = 5.38; VigiBase = 9.46)
- ‘Autoimmune hepatitis’ (PRRs: FAERS = 35.9; VigiBase = 60.3), ‘Hepatitis’ (PRRs: FAERS = 9.76; VigiBase = 2.9), and ‘Hepatotoxicity’ (PRRs: FAERS = 4.65; VigiBase = 7.73)
- ‘Pneumonitis’ (PRRs: FAERS = 21.44; VigiBase = 37.14)
- ‘Myasthenia gravis’ (PRR: FAERS = 21.46)
- ‘Type 1 diabetes mellitus’ (PRRs: FAERS = 18.63; VigiBase = 34.90) and ‘Diabetic ketoacidosis’ (PRRs: FAERS = 4.3; VigiBase = 9.4)
- ‘Uveitis’ (PRRs: FAERS = 15.51; VigiBase = 35.22)
- ‘Myocarditis’ (PRR: FAERS = 12.54)
- ‘Myositis’ (PRR: FAERS = 10.23)
- ‘Tubulointerstitial nephritis’ (PRRs: FAERS = 5.66; VigiBase = 7.69)

Our analysis captured, in addition, some other reactions that referred to general patient conditions or that were more difficult to classify. These include ‘Asthenia’ (VigiBase), ‘Decreased appetite’ (FAERS, VigiBase), ‘Dehydration’ (VigiBase), ‘Fatigue’ (FAERS, VigiBase), ‘General physical health deterioration’ (FAERS, VigiBase), ‘Pyrexia’ (FAERS), ‘Sepsis’ (VigiBase), ‘Weight decreased’ (VigiBase). Infections, inflammations and related side effects such as pyrexia or eczema were ambiguous as they could be either symptoms of an infusion reaction or signs of an infection, as well as ‘Blood bilirubin increased’ (FAERS, VigiBase) and ‘Hyponatraemia’ (FAERS, VigiBase) might represent both hepatic and/or hematologic effects.

We also observed that some irAEs occur in absolute numbers more frequently in women, such as arthritis, pancreatitis, pneumonitis, uveitis, vitiligo, autoimmune colitis, or hyperthyroidism. However, the examined PembroM cohorts contained more male patient cases, AEs with several missing values, and the data were not normalized in this respect. 

### 3.3. Comparison of Pembrolizumab, Ipilimumab and Nivolumab Monotherapies in Melanoma

We isolated the AEs of melanoma patients from FAERS and VigiBase that reported only Pembrolizumab and no other drugs (cohorts named ‘Pembrolizumab (alone)’), and compared them with results from similar Ipilimumab and Nivolumab cohorts of previous studies. Table 3 contrasts reaction PTs mentioned in more than 1% of the FAERS ‘Pembrolizumab (alone)’ cohort with summarized FAERS data for Ipilimumab (2704 AEs) and Nivolumab (890 AEs) in melanoma [33].

However, Table 3 should not serve as a direct comparison due to relative differences such as the number of reactions reported in each cohort, as well as the reduced number of occurrences observed for each agent-reaction combination in those smaller cohorts. Additional information for all reactions reported in these cohorts can be found in our supplementary files and in that of [33].

Therefore, Table 3 lists only key reactions for the ‘Ipilimumab (alone)’ and ‘Nivolumab (alone)’ cohorts, extracted from [33]. Furthermore, Table 3 does not mention all reactions observed for each category and therefore ‘empty’ cells (with no terms) do not necessarily represent absence of respective phenotypes from a cohort, but rather an occurrence in less than 1% of that cohort’s cases, or non-statistically significant observations. In addition, our arbitrary cut-off threshold of one percent for each cohort’s size refers to a different amount of cases every time. In turn, the terms ‘Hypophysitis’ and ‘Hyponatremia’, for example, are not mentioned for the ‘Nivolumab (alone)’ cohort (despite their statistically significant PRR signals 114.42 and 2.44, respectively) because they were reported in less than one percent of its cases, while ‘Anaemia’ (occurring in > 1% of its cases, with PRR 1.17) was not found to be statistically significant. Similarly, the term ‘Uveitis’ is not listed for the ‘Ipilimumab (alone)’ cohort (with statistically significant PRR value of 12.26) as it was mentioned in less than one percent of this cohort’s cases. Accordingly, for the FAERS ‘Pembrolizumab (alone)’ cohort, there are reactions reported in < 1% of its cases that are related to adrenal insufficiency and (autoimmune) hepatitis conditions, for example. Last, Table 3 highlights also some observations from the VigiBase ‘Pembrolizumab (alone)’ cohort denoting signal variations between the two (FAERS and VigiBase) ‘Pembrolizumab (alone)’ cohorts attributed to differences in the two data sets’ contents and release dates.

Overall, the three ICIs have both related side effects but also distinct profiles with adrenal, blood, gastrointestinal, hepatic, hypothalamic, renal, respiratory, skin and thyroid dysfunctions being the main safety concerns.

## 4. Discussion

To examine the safety of Pembrolizumab in the treatment of melanoma, we reviewed patient safety data extracted from AE repositories. Ongoing clinical trials on Pembrolizumab are on a par with other ICI studies that investigate novel combination therapies and strategies or focus on more specific settings, such as on poor prognosis groups or on refractory metastatic patients [34]. However, the prognostic and/or predictive value of numerous immunotherapy biomarker candidates has not been systematically validated yet in prospective clinical trials, and their utility for guiding a treatment decision needs further investigations. Moreover, one key aspect during ICI therapy monitoring involves the early identification and management of the—sometimes, irreversible—irAEs. As molecular biomarkers for irAEs are also limited, it is unclear how to deal with potential patient characteristics or findings, and several efforts focus on summarizing ICI toxicities and on developing guidelines for the management of irAEs (e.g., [15,20,24]). Knowing more about potential risk factors and being aware of the types of AEs associated with specific ICIs might help to more efficiently recognize and treat irAEs. This is of great importance considering the results of a recent study in mice xenografted with human colon cancer cells, suggesting that tumor necrosis factor (*TNF*) inhibition combined with *CTLA-4* and *PD-1* immunotherapy may provide a clinically feasible prophylactic strategy regarding some irAEs [35,36].

One other aspect pertaining to irAEs is that their emergence has been suggested to correlate with clinical benefit to the immunotherapeutic agents, implying a potential predictive role for response to ICI treatment [37,38,39]. This is particularly important considering the limited number of both biomarkers with definitive predictive value in melanoma immunotherapy and alternative treatment options in the malignancy. However, further studies are needed to confirm this, highlighting the importance of AE profiling efforts based on evidence from real world event observations.

We therefore processed publicly available AE cases to extract reactions of melanoma patients treated with Pembrolizumab (Table 1 and Table 2) and compared with respective toxicity profiles of Nivolumab and Ipilimumab from FAERS (Table 3). Our analysis includes real world events and provides additional insight to previous safety profiling efforts that were based on translational studies, clinical trials, and meta-analyses. Our results also provide an update to similar AE-based studies that confirmed known safety considerations [23,40]. Last, our work is an advance over previous studies in that it examined Pembrolizumab AEs for more melanoma patients, and in that it considered AE cases extracted from different spontaneous AE repositories (FAERS and VigiBase).

We detected irAEs that affect a variety of organs/systems, including endocrine, dermatologic, gastrointestinal, hepatic, pulmonary, neurological, cardiac, renal, hematologic and musculoskeletal complications, as well as infusion/infection reactions. Comparing Pembrolizumab’s FAERS AE profile in melanoma with those of Nivolumab and Ipilimumab showed that, despite their somewhat distinct profile, Pembrolizumab and Nivolumab were more similar to each other than to Ipilimumab. For example, colitis, hypophysitis and rash were reported in larger proportions of the Ipilimumab cohort, while pneumonitis, hypothyroidism, arthralgia or vitiligos were reported more frequently with Pembrolizumab or Nivolumab. This finding is in line with results of clinical trials and is supported by the similar mechanism of action of these agents as *PD-1* inhibitors, compared to that of the *CTLA-4* inhibitor Ipilimumab [41].

In our PembroM AE datasets from FAERS and VigiBase, several co-medications, which have been plausibly used for the treatments of side effects or the prevention of AE conditions, were reported. For example, antihistamines were most likely prescribed to alleviate rash or pruritus. This information is also captured by several of the drugs’ indications that reflected AE reactions. Characteristically, while the most frequent Anatomical Therapeutic Chemical (ATC) Classification System category of level 2 (i.e., pharmacological or therapeutic subgroup) by number of PembroM FAERS cases was ‘analgesics’, mentioned in 15.58% cases (357 AEs) of this cohort, the top level 2 ATC class by PRR score was ‘thyroid therapy’ mentioned in 6.98% cases (160 AEs). However, without knowledge of the patients’ prior history, no robust conclusions can be drawn regarding incidence or potential interactions between Pembrolizumab and these medications. Nevertheless, with approximately 40% of reports containing co-medication information, the PembroM cohorts provide a useful dataset for performing comparative co-administration analyses and the generation of drug interaction hypotheses, particularly in the context of other co-morbidities. However, we did not pursue this direction further, due to the reduced numbers of such AEs contained in the examined PembroM cohorts, though we believe that this will improve over time as more reports become available. Furthermore, a few cases reported also the administration of Nivolumab or Ipilimumab. The reporting of these two immunotherapeutic agents most likely represents a therapy switch between the different ICI treatments, since the only ICI combination approved by the US FDA for the treatment of advanced melanoma is that of Ipilimumab plus Nivolumab.

It is important to note here that our results should not be interpreted as calculated incidences in the general melanoma patient population, as the reported frequencies represent occurrence in patients who manifested AEs during therapy and their incidence was reported. This is also not the intended use of data coming from spontaneous AE repositories since they contain only AEs and are therefore biased without proper normalization considering reference/control data. 

Moreover, there is a number of parameters of high clinical significance that would additionally benefit our study and the assessment of ICI pharmaceuticals [42]. However, we could not consider data that are neither available nor straightforward to extract from public AE repositories. Some such factors that could not be yielded for our analysis include detailed patient/event history (including treatment duration, timing, dosage or previous therapies and co-morbidities), the setting in which the treatment took place (e.g., first-line systemic treatment in patients with unresectable melanoma or adjuvant treatment after tumor resection), patient Karnofsky scores or Eastern Cooperative Oncology Group (ECOG) performance status, the duration of irAEs, or specific AE severity grading. 

One other major issue related to irAEs is the medical costs associated with them. In this context, up-to-date knowledge about these side effects remains a critical issue for the healthcare system, as well as for many patients who might suffer poorer quality-of-life without any tangible clinical benefit from specific drug treatment(s). Adoption of integrated safety assessment and interpretation techniques is potentially a key factor to help understand better the landscape underlying such economic costs of AEs associated with ICIs, providing the opportunity to shift the tradeoff between AE costs and the clinical benefit derived from the use of these agents towards the best for patients and for the community.

Computational AE analytics provide informative clinical insights for this goal and an augmented resource for the systematic characterization of safety considerations (e.g., [33,43,44]), especially when combined with additional data dimensions such as molecular parameters (e.g., [30,45,46,47]). As more real world data emerges we anticipate future directions to include characterization of data from more AERS (e.g., EudraVigilance), more informed prospective therapy studies, improved irAE diagnosis and characterization methods, investigation of tumour-dependent irAE profiles, and identification of patient subgroups with little benefit from ICI therapy so as to avoid unnecessary irAE occurrence. Our results thus provide important additional insights to support the current global efforts to characterize the utility and safety of Pembrolizumab in melanoma.

## 5. Conclusions

Based on evidence extracted from the public FAERS and VigiBase repositories, we inspected two sets of AEs, each with more than two thousand melanoma patients treated with Pembrolizumab. Our results confirm known toxicological considerations for this, and other widely used, immunotherapeutic agents’ related and distinct side-effect profiles, and identify specific immune-related reactions from different organs and systems as well as infusion/infection reactions. Our study provides additional insight to the profiling efforts of ICIs and we expect that AE awareness and knowledge on potential risk factors will help in the early recognition and informed management of irAEs in melanoma patients treated with ICIs.

## Figures and Tables

**Table 1:** Frequent MedDRA preferred term (PT) reactions (level 4 terms) mentioned in both PembroM cohorts. All observations listed are statistically significant, with *p*-value <0.05.

Reaction PT Name ^1^	FAERS (PembroM Cohort)			VigiBase (PembroM Cohort)		
	AEs	% Cohort	PRR	AEs	% Cohort	PRR
Alanine aminotransferase increased	26	1.13	2.59_p_	23	0.92	2.58_p_
Anaemia ^2^	52	2.27	1.97*_p_*	47	1.87	2.18*_p_*
Arthralgia ^2^	75	3.27	1.69_p_	81	3.23	2.18*_p_*
Arthritis	16	0.69	1.75	19	0.76	2.76
Aspartate aminotransferase increased	27	1.18	2.99*_p_*	22	0.88	3.01*_p_*
Autoimmune hepatitis ^2 +^	27	1.18	35.83 *_p_*	23	0.92	60.29 *_p_*
Blood bilirubin increased	12	0.52	2.61	11	0.44	3.96
Colitis ^+^	47	2.05	13.09*_p_*	60	2.39	22.12*_p_*
Constipation	37	1.62	1.59	44	1.76	1.95_p_
Decreased appetite ^2^	53	2.3	1.83_p_	70	2.79	2.71*_p_*
Diabetes mellitus	22	0.96	1.87	16	0.64	2.35
Diabetic ketoacidosis ^+^	11	0.48	4.29_p_	14	0.56	9.41*_p_*
Diarrhoea ^2^	104	4.54	1.53_p_	129	5.15	1.78*_p_*
Dry mouth	18	0.79	1.71	22	0.88	1.56
Dry skin	13	0.57	1.98	15	0.59	2.97
Dyspnoea exertional ^+^	11	0.48	3.01	17	0.68	8.82*_p_*
Fatigue ^2^	202	8.82	2.42*_p_*	248	9.89	3.80*_p_*
General physical health deterioration ^+^	25	1.09	2.09	26	1.04	5.05*_p_*
Hepatic enzyme increased	15	0.65	2.02	19	0.76	2.39
Hepatitis ^+^	33	1.44	9.76*_p_*	20	0.79	2.89_p_
Hepatotoxicity ^+^	10	0.44	4.65_p_	10	0.39	7.73*_p_*
Hyperglycaemia	16	0.69	3.11_p_	18	0.72	3.20_p_
Hyperthyroidism ^+^	11	0.48	7.16*_p_*	38	1.52	23.94*_p_*
Hyponatraemia	31	1.35	4.20*_p_*	27	1.08	3.92*_p_*
Hypophysitis ^2 +^	34	1.48	267.83 *_p_*	26	1.04	308.83 *_p_*
Hypothyroidism ^2 +^	41	1.79	11.22*_p_*	74	2.95	30.35*_p_*
Interstitial lung disease	20	0.87	3.74*_p_*	16	0.64	4.61*_p_*
Lung disorder ^+^	15	0.65	2.61	14	0.56	5.13*_p_*
Lymphadenopathy	12	0.52	2.58	15	0.59	2.54
Muscular weakness	30	1.31	1.95	30	1.19	2.51*_p_*
Myalgia	33	1.44	1.47	43	1.72	1.41
Pancreatitis	18	0.79	2.14	16	0.64	2.59
Pemphigoid ^2 +^	14	0.61	25.48 *_p_*	23	0.92	53.54 *_p_*
Pleural effusion	17	0.74	1.95	19	0.76	4.29*_p_*
Pneumonitis ^2 +^	54	2.36	21.44 *_p_*	68	2.71	37.14 *_p_*
Rhabdomyolysis	12	0.52	1.99	10	0.39	2.21
Thyroid disorder ^+^	10	0.44	5.38_p_	10	0.39	9.46*_p_*
Thyroiditis ^2 +^	10	0.44	42.42 *_p_*	17	0.68	81.45 *_p_*
Tubulointerstitial nephritis ^+^	10	0.44	5.66_p_	10	0.39	7.69*_p_*
Type 1 diabetes mellitus ^+^	16	0.69	18.63*_p_*	17	0.68	34.90*_p_*
Uveitis ^+^	15	0.65	15.51*_p_*	25	0.99	35.22*_p_*
Vitiligo ^2 +^	26	1.13	166.64 *_p_*	84	3.35	373.84 *_p_*

^1^ PTs are alphabetically sorted. Listed are only those PTs that are mentioned in both FAERS and VigiBase PembroM cohorts, reported in more than ten AEs in each, and with PRR > 1 in both cohorts. For this reason, similar reactions reported with different PTs in the two cohorts are not included, despite their statistical strength. Such examples include PTs like ‘Asthenia’, ‘Autoimmune colitis’, ‘Hypopituitarism’, ‘Myasthenia gravis’, ‘Myocarditis’, ‘Myositis’, ‘Pyrexia’, ‘Pruritus’, ‘Rash’, ‘Rash generalized’, ‘Renal failure acute’, etc. ^2^ Underlined results highlight the six highest values, per metric and set. ^+^ Indicates PRR > 5 in either cohort. Subscript _p_ denotes 0.0001 ≥ *p*-value > 0.00001 while subscript *_p_* refers to *p*-values ≤ 0.00001.

**Table 2:** Most frequently MedDRA preferred term (PT) reactions (level 4 terms) reported with Pembrolizumab in Melanoma. All observations listed are statistically significant, with *p*-Value < 0.05.

Organ/System Class ^1^	Reaction PT Name ^(whether FAERS and/or VigiBase PembroM cohort) 2^
Brain, neurologic	‘Cerebral haemorrhage’ ^F^, ‘Myasthenia gravis’ ^F^
Cardiac	‘Myocarditis’ ^F^
Endocrine	‘Adrenal insufficiency’ ^FV^, ‘Hyperglycaemia’ ^FV^, ‘Hyperthyroidism’ ^FV^, ‘Hypophysitis’ ^FV^, ‘Hypopituitarism’ ^F^, ‘Hypothyroidism’ ^FV^, ‘Thyroid disorder’ ^FV^, ‘Thyroiditis’ ^FV^, ‘Diabetes mellitus’ ^FV^, ‘Diabetic ketoacidosis’ ^FV^, ‘Type 1 diabetes mellitus’ ^FV^
Gastrointestinal	‘Autoimmune colitis’ ^V^, ‘Colitis’ ^FV^, ‘Constipation’ ^FV^, ‘Diarrhoea’ ^FV^, ‘Dry mouth’ ^FV^
Hematologic, vascular	‘Anaemia’ ^FV^, ‘Eosinophilia’ ^F^, ‘Peripheral swelling’ ^V^
Kidney, renal	‘Acute kidney injury’ ^V^, ‘Blood creatinine increased’ ^V^, ‘Renal failure acute’ ^F^, ‘Tubulointerstitial nephritis’ ^FV^
Liver, hepatic	‘Alanine aminotransferase increased’ ^FV^, ‘Aspartate aminotransferase increased’ ^FV^, ‘Autoimmune hepatitis’ ^FV^, ‘Hepatic enzyme increased’ ^FV^, ‘Hepatitis’ ^FV^, ‘Hepatocellular injury’ ^V^, ‘Hepatotoxicity’ ^FV^, ‘Transaminases increased’ ^V^
Lymphadenopathies	‘Lymphadenopathy’ ^FV^
Musculoskeletal	‘Arthralgia’ ^FV^, ‘Arthritis’ ^FV^, ‘Muscular weakness’ ^FV^, ‘Myalgia’ ^FV^, ‘Myositis’ ^F^, ‘Rhabdomyolysis’ ^FV^
Opthalmologic	‘Uveitis’ ^FV^
Pancreatic	‘Lipase increased’ ^V^, ‘Pancreatitis’ ^FV^
Respiratory, pulmonary	‘Cough’ ^V^, ‘Dyspnoea exertional’ ^FV^, ‘Interstitial lung disease’ ^FV^, ‘Lung disorder’ ^FV^, ‘Pleural effusion’ ^FV^, ‘Pneumonitis’ ^FV^, ‘Pulmonary embolism’ ^V^
Skin	‘Dry skin’ ^FV^, ‘Eczema’ ^V^,’Pemphigoid’ ^FV^, ‘Pruritus’ ^F^, ‘Psoriasis’ ^V^, ‘Rash’ ^F^, ‘Rash erythematous’ ^F^, ‘Rash generalised’ ^V^, ‘Rash maculo-papular’ ^F^, ‘Vitiligo’ ^FV^

^1^ Results are presented in alphabetical order of organ/system group. ^2^ Reaction MedDRA PT names are listed in alphabetical order. Superscript symbols ^F^, ^V^, and ^FV^ denote FAERS, VigiBase, and both FAERS and VigiBase PembroM cohorts, respectively. The table contains reaction PTs that have PRR > 1, that are statistically significant, and that occur in at least ten AEs of the respective cohort.

**Table 3:** Summary of adverse reactions reported in FAERS for Melanoma patients treated with Pembrolizumab alone, or with Ipilimumab or Nivolumab only. The table contrasts frequent reactions in these cohorts—full datasets are available as supplementary files (see also [33]). Superscript numbers in parentheses indicate respective PRR values from each FAERS cohort; all values presented are statistically significant.

Category/Class	Pembrolizumab (alone) ^1^	Ipilimumab (alone) ^2^	Nivolumab (alone) ^2^
Adrenal	^(+)^	Adrenal insufficiency ^(42.38)^	Adrenal insufficiency ^(27.29)^
Blood **^3^**	Anaemia Hyponatremia ^(3.54)^	Anaemia ^(1.48)^ Hyponatremia ^(4.02)^	^(+)^
Febrile	Pyrexia	Pyrexia ^(2.03)^	^(+)^
Gastrointestinal **^3^**	Colitis ^(10.45)^ Diarrhoea	Colitis ^(72.05)^ Diarrhoea ^(4.85)^ Intestinal Perforation ^(10.74)^	Colitis ^(12.88)^ Diarrhoea ^(1.44)^
Hepatic	^(+)^	Hepatitis ^(9.02)^	Hepatic function abnormal ^(8.45)^ Liver disorder ^(3.93)^
Hypothalamic	Hypophysitis ^(210.73)^	Hypophysitis ^(1051.12)^	^(+)^
Opthalmologic **^3^**	^(+)^	^(+)^	Uveitis ^(26.59)^
Renal	^(+)^	Renal Failure Acute ^(1.97)^	Acute Kidney Injury ^(2.27)^
Respiratory	Pneumonitis ^(18.11)^	Pneumonitis ^(11.07)^	Pneumonitis ^(16.28)^
Skin **^3^**	Pruritus ^(1.77)^ Rash ^(1.69)^	Pruritus ^(1.85)^ Rash ^(3.29)^	Leukoderma ^(2439.24)^ Pruritus ^(2.07)^
Thyroid **^3^**	Hypothyroidism ^(8.48)^	Hypothyroidism ^(7.88)^	Hypothyroidism ^(29.59)^
Other	Arthralgia ^(1.51)^ Decreased Appetite ^(1.58)^ Constipation Myalgia	Decreased Appetite ^(2.49)^ Dehydration ^(3.43)^ Sepsis ^(2.22)^	Decreased Appetite ^(2.15)^ Infusion Related Reaction ^(4.41)^ Sepsis ^(1.95)^

^1^ MedDRA preferred terms (PTs) of reactions reported in more than 1% of the FAERS ‘Pembrolizumab (alone)’ cohort’s AEs; the following terms were excluded: ‘Malignant neoplasm progression’, ‘Death’, ‘Fatigue’, ‘Adverse event’, ‘Nausea’, ‘Disease progression’, ‘Drug ineffective’, ‘Headache’, ‘Dyspnoea’, ‘Asthenia’, ‘Pain’, ‘Vomiting’, ‘Weight decreased’, ‘Metastatic malignant melanoma’, ‘Malaise’; PTs without PRR scores noted represent non-statistically significant observations (i.e., with p > 0.05). ^2^ Data adapted from the results of Figure 3 of [33]; original names of respective cohorts were ‘Ipilimumab (only)’ and ‘Nivolumab (only)’. ^3^ The VigiBase ‘Pembrolizumab (alone)’ cohort reported ‘Anemia’ (PRR: 1.68; *p* < 0.05), ‘Colitis’ (PRR: 18.9; *p* < 0.05), ‘Diarrhoea’ (PRR: 1.37; *p* < 0.05), ‘Uveitis’ (PRR: 36.12; *p* < 0.05), ‘Vitiligo’ (PRR: 452.34; *p* < 0.05), and ’ Hyperthyroidism’ (PRR: 28.52; *p* < 0.05) in more than 1% of its AEs. ^(+)^ For additional reaction terms occurring in each cohort’s cases, see respective Supplementary Files.

## Supplementary Materials

The following are available online at https://www.mdpi.com/2072-6694/12/4/1008/s1, Supplementary file: Summarized views of the examined adverse event cohorts.
